# Spiking burstiness and working memory in the human medial temporal lobe

**DOI:** 10.1093/texcom/tgac039

**Published:** 2022-10-19

**Authors:** Francesco Cocina, Andreas Vitalis, Amedeo Caflisch

**Affiliations:** Department of Biochemistry, University of Zurich, Winterthurerstrasse 190, CH-8057 Zurich, Switzerland; Department of Biochemistry, University of Zurich, Winterthurerstrasse 190, CH-8057 Zurich, Switzerland; Department of Biochemistry, University of Zurich, Winterthurerstrasse 190, CH-8057 Zurich, Switzerland

**Keywords:** amygdala, decoding, hippocampus, memory workload, persistent activity

## Abstract

Persistent activity has commonly been considered to be a hallmark of working memory (WM). Recent evidence indicates that neuronal discharges in the medial temporal lobe (MTL) are compatible with WM neural patterns observed in cortical areas. However, the characterization of this activity rarely consists of measurements other than firing rates of single neurons. Moreover, a varied repertoire of firing dynamics has been reported in the MTL regions, which motivate the more detailed examination of the relationships between WM processes and discharge patterns undertaken here. Specifically, we investigate‚ at different resolution levels, firing irregularities in electrode recordings from the hippocampus, amygdala, and the entorhinal cortex of epileptic patients during a WM task. We show that some types of (ir)regularities predict response times of the patients depending on the trial periods under consideration. Prominent burst activity at the population level is observed in the amygdala and entorhinal cortex during memory retrieval. In general, regular and bursty neurons contribute to the decoding of the memory load, yet they display important differences across the three anatomical areas. Our results suggest that nonrandom (non-Poisson) patterns are relevant for WM, which calls for the development and use of statistics complementary to mere spike counts.

## Introduction

A great deal of work has been dedicated over the last 50 years to the identification and characterization of neural activity observed during working memory (WM) tasks (Fuster and Alexander 1971; Wang 2021). The ability to maintain information in memory for a limited period of time has been traditionally explained in terms of biological mechanism by the presence of persistent spiking activity during the maintenance, or delay, period of the task. The term ‘persistent’ has been used in association with the elevated spike count of a restricted subset of neurons (Constantinidis et al. 2018; Leavitt et al. 2017; Wang 2021) as well as to describe low-dimensional neural trajectories residing in an attractor state (Kamiński et al. 2017; Masse et al. 2020). The brain areas typically associated to WM are the prefrontal, parietal, and sensory cortex (Christophel et al. 2017). However, recently, also the medial temporal lobe (MTL) has attracted attention following the identification of signatures of persistent activity (Bausch et al. 2021; Kamiński et al. 2020; Kornblith et al. 2017) (see also earlier work in Axmacher et al. (2007)).

Despite the multiple reports, a precise characterization of the persistent activity in terms of firing dynamics is missing. In addition, the properties of this activity that allow for an efficient coding of the memory variables are not fully understood. Here, we ask the question if bursty neurons and/or population bursts contribute to the decoding of WM signals. To answer this question, we investigate firing irregularities in the MTL during a modified Sternberg WM task. We utilize a data set of recordings from a region comprising the hippocampus, amygdala, and the entorhinal cortex of epileptic patients (available online Boran et al. 2019; 2020). In the original work (Boran et al. 2019), the authors hypothesized the involvement of the hippocampus in the WM process, related in particular to the memory load, and they used sets of 4, 6, or 8 letters written on a screen to be memorized. These authors found two sets of neurons with significantly enhanced activity during the delay phase and the memory retrieval phase, which were termed maintenance and probe neurons, respectively. An attractor-driven dynamics was postulated for the maintenance of WM information at the population level.

Temporally irregular neural activity was previously observed in the prefrontal cortex during the delay period of a delayed response task (Compte et al. 2003). Furthermore, large deviations in inter-spike interval (ISI) statistics from a Poisson process were unveiled across the cortex with differential patterns from sensory-motor to higher cortical regions (Maimon and Assad 2009). In the human MTL, long-range temporal correlations among the ISIs were observed in both amygdala and hippocampus in spontaneous activity (Bhattacharya et al. 2005). More recently, a wealth of distinct irregular patterns associated with temporal coding during different memory phases was identified in the hippocampus and entorhinal cortex (Umbach et al. 2020).

In contrast to the original analysis, we here do not focus exclusively on the firing rates of single neurons but rather investigate the discharge patterns in terms of burstiness, i.e. irregularity of the spike sequences. In particular, we analyze the spike trains by adopting, predominantly, a local variation metric (Shinomoto et al. 2003; 2009) for the quantification of irregularities, which is independent from raw firing rates as we demonstrate below. Our analysis is structured as follows: first, we validate the local variation metric and investigate if it can be reliably related to trial and behavioural variables; second, we examine the burst activity at the population level and assess how it reports on memory processes and on the coordination of single units; a Fano factor analysis then helps clarify some of the previous results. Eventually, we study how the population decoding of trial variables depends on the firing behaviour of the underlying units. Our analysis sheds light on intrinsic differences between the dynamics of the 3 anatomical areas. It also reveals the relevance of nonrandom (non-Poisson) spike trains for memory maintenance and behavioural performance, i.e. response times.

## Materials and methods

### Experimental design and recordings

Detailed information about subjects, task, recording setup, and spike sorting procedure can be found in Boran et al. (2020). All subjects provided written informed consent for the study. In this section, we recapitulate the aspects that strictly concern our analysis and describe the data filtering procedure. Subjects are asked to perform a modified Sternberg task, which comprises epochs of encoding, maintenance, and recall of memory items. In detail, after an initial period of fixating a blank screen (1 s), a stimulus is presented. This consists of a set of central 4, 6, or 8 letters, possibly surrounded by ‘X’s on both sides such that the number of characters is always equal to 8. The letter ‘X’ is never part of the set to be memorized. The encoding period lasts for 2 s and is followed by a maintenance, or delay, period of 3 s where a blank screen is shown again. A probe letter is eventually presented and the subjects are instructed to answer rapidly whether the letter does or does not belong to the stimulus set (IN or OUT button press). The set sizes are randomly selected, except when the subject response is wrong. In that case, the set size of the subsequent trial is always chosen as 4 in order to keep the patient motivated. A session is composed of 50 trials and lasts approximately 10 min.

Depth electrodes were implanted in the MTL of epileptic patients for potential surgical resection of their seizure foci. Intracranial electroencephalography recordings were performed into hippocampus, amygdala, and the entorhinal cortex with depth electrodes combining macro- and microcontacts (1.3-mm diameter, 8 macrocontacts of 1.6-mm length, spacing between contact centers 5mm, 9 microcontacts protruding radially 4mm from its tip, ADTech^®^). All of the trial recordings have a fixed length of 8 s and they all start with the fixation period (1 s). This implies that when response times are longer than 2 s a part of the neural recordings preceding the response is missing. In many cases, the trials affected are discarded; this is properly noted where required. Trials containing artifacts were also not considered throughout the analysis (Boran et al. 2019).

Spike sorting has been performed through the Combinato package (Niediek et al. 2016), and its results are provided with the data set. In our analysis, only neurons with an average firing rate >1 Hz across trials were kept. Also, to control for potential cross-talk between electrodes, Jaccard similarity was computed between binarized spike trains (1-ms bin) of simultaneously recorded units. All of the session trials were concatenated for the calculation. Values of Jaccard similarity higher than 0.3 were considered suspicious, and sequentially selected units were discarded until all values fell below this threshold. In detail, we identified the unit that was contributing to the highest number of Jaccard values >0.3 and removed it. If 2 units were equally contributing, we discarded the one with lower firing rate. The procedure was repeated until the threshold criterion was fulfilled globally. In the end, 7 out of 26 sessions were affected with a total number of discarded units equal to 35 (out of 992 previously selected).

It is known that bursting activity can compromise the identification of single units during spike sorting (Quirk and Wilson 1999). This is mostly due to the distortion of the spike waveform following sustained firing, and sorting of the spike shapes can be further complicated when multiple bursty neurons are recorded from the same electrode (Einevoll et al. 2012; Lewicki 1998). As a consequence, the distribution of units with respect to their burstiness values (see below) might depend on the chosen spike sorting technique (Sukiban et al. 2019). The original authors evaluated quality metrics and manually curated the putative clusters (Boran et al. 2020). However, it is reasonable to expect some variability with respect to our results deriving solely from the chosen sorting procedure.

### Firing irregularities

#### $LvR$ metric

Spiking irregularities were investigated by examining the sequence of the ISIs. We adopted an enhanced local variation measure ($LvR$) to quantify the firing (ir)regularities of the units during single trials (Shinomoto et al. 2003; 2009). Unlike other common metrics, such as the coefficient of variation, $LvR$ accounts for fluctuations in firing rates along the time series and, also, corrects for the refractory period following a spike. It is computed as

(1) \begin{align*}& LvR = \frac{3}{n-1}\sum_{i=1}^{n-1}\left(1-\frac{4I_{i}I_{i+1}}{(I_i + I_{i+1})^2}\right)\left( 1 + \frac{4R}{I_i + I_{i+1}}\right), \end{align*}

where $I$ indicates the ISI, $n$ the total number of ISIs, and $R$ the refractoriness constant. This latter parameter is set to 5 ms (as in the original work of Shinomoto et al. (2003)) for the single-unit calculations. For the combination of two or more units, the refractoriness correction was not considered ($R$ = 0). $LvR$ values were computed for (windows of) spike sequences containing at least 5 spikes. When this condition was not met, the corresponding data points were simply discarded unless noted otherwise.

#### Change points

We identified sharp variations in the single-unit firing rates during each trial through an adaptive CP procedure (Gallistel et al. 2004; Jezzini et al. 2013). The method uses the empirical cumulative count of spikes and compares it with the expected one, which, in our case, is the one deriving from a perfectly regular firing with a matched number of spikes (i.e. a uniform distribution). The earliest time point where the two distributions differ maximally is considered and identified as a CP contingent upon the result of a binomial test between the spike counts before and after that point (see Gallistel et al. (2004) for more details). After a CP is evaluated to be significant, the algorithm is applied again to the remaining data following the CP. The adaptive element of the procedure rests in reducing progressively the $P$-value confidence threshold until no change points (CPs) are identified during the fixation period (Jezzini et al. 2013). This is done in practice by increasing the logit = $\log \frac{1-P}{P}$ in steps of 0.2 from 1.3 to 5.9 (corresponding to a range $P\simeq 0.05-10^{-6}$).

### Time-resolved analysis of irregularities and trial variables

To investigate the relation between $LvR$ and response times, we calculated the Pearson correlations between these 2 quantities using data from the whole pool of sessions and trials (Fig. 4). Differently, in order to assess whether there are significant regions in the $LvR$ spectrum tuned to a particular trial class, i.e. set size or response correctness (Fig. S3), we devised the following test. The $LvR$ values are first collected across all of the sessions and trials as above and transformed into ranks following an ascending order (1 was assigned to the lowest $LvR$, 2 to the second-lowest, and so on). This was done separately for single as well as combined (2 and 3 units, see Fig. 3a) $LvR$ values. For the combined case, we subsampled only a fraction of points (see below). The cumulative distributions of the rank-transformed $LvR$ values are then computed for the single classes, e.g. correct and wrong responses. The rank transformation makes the (following) statistic invariant to the underlying distributions (see Fig. S2a) and guarantees a fair comparison between the different combinations. We took the sum of the point-wise differences between the 2 curves as the test statistic for quantifying the dissimilarity between the 2 distributions. For the set size grouping variable, the difference was between high (6 and 8) and low workloads (4); for the response correctness, we considered the difference between the wrong and correct subsets. For this latter grouping variable, we considered only sessions with at least 5 wrong trials. The statistic obtained is equal to the total area between the 2 curves but respecting the sign of the difference. It differs from the more known Wasserstein, or earth-mover’s, distance as there the absolute difference between the curves is considered.

#### Subsampling of combinations and statistical testing

Next, we describe the procedures for subsampling the data set and calculating the chance level distribution which are conceptually the same also for both of the analyses introduced before. In order to maintain across the different unit combinations (single, 2, or 3) the same number of points used for calculating the test statistic (the Pearson correlation or the dissimilarity between the class distributions), we subsampled, within each session, from the combined $LvR$ values (2 and 3) a number of points equal to the ones in the single-unit calculation. From this, the test statistic is computed, and the whole process is repeated 100 times. Within each repetition (and also for the single-unit $LvR$), we generated a null distribution by permuting 1,000 times the trial variables, i.e. response times, set sizes, or correct/wrong responses. This permutation was performed preserving both the trial structure and the subject identity, that is, units that were simultaneously recorded were assigned the same trial variable, drawn from the ones available for that specific subject. By comparing the true statistic value to the null distribution, a $P$-value could be extracted. The summary $P$-value for the combined $LvR$ is given by the median of the 100 extracted ones.

### Population bursts

A population burst is defined as a period of sustained collective activity where the population firing rate exceeds a certain threshold for at least 100 ms. Our procedure closely resembles the one in Vaz et al. (2020). Instantaneous firing rates were extracted by convolving the spike rasters of each unit with a Gaussian kernel of bandwidth equal to 25 ms and subsampling with a 10-ms step. These time series were used for calculating both the population firing rate, as the average value across the units, and the threshold, which is described in the following. In turn, (i) single-unit firing rates were averaged over the whole trial window; (ii) these resulting values were then averaged across units within each trial; and (iii) the mean and standard deviation across the trials were extracted. These last 2 values eventually served to define the threshold as mean $+$ 3$\,\cdot$ s.d. If two consecutive population bursts were closer than 150 ms (tail to head), the one with lower firing rate was discarded. We assigned the single burst events to a specific trial period if at least 80% of the burst window was located within its boundaries. When dealing with the probe period, the right boundary was always defined by the response time.

Below, we refer to some of the analyses presented in the Supplementary Information. The unit composition of a single population burst, $\boldsymbol{w}$, was defined as a vector composed by the averages of the single-unit firing rates within the burst window. The smoothed firing rates (25-ms Gaussian kernel) were again employed. The sparsity measure quantifies the prevalence of a group of units in the population burst activity. It is computed as follows:

(2) \begin{align*}& 1 - \frac{\sqrt{n} - \sum_i \lvert \hat{w}_i\rvert}{\sqrt{n} - 1}, \end{align*}

where $n$ is equal to the number of units (the length of $\boldsymbol{w}$), and $\hat{\boldsymbol{w}}$ is the unit composition scaled to unit length. Sparsity values close to zero indicate the net prevalence of few units to the population burst activity, whereas higher values suggest a more balanced contribution of all of the units. The $LvR$ of the population burst events was calculated as the weighted mean of the single-unit $LvR$ using the unit composition elements $\boldsymbol{w}$ as weights.

### Fano factor

Mean-matched Fano factors (FF) were calculated to investigate the trial variability of the spiking responses (Churchland et al. 2010). In contrast to the raw FF calculation, that is, the simple ratio between variance and mean of the spike counts, the mean-matched procedure controls for local variations in firing rates that can trivially affect the FF calculation. For example, similar to $LvR$, the statistic should account for the fact that refractoriness periods might reduce the spiking variability in periods of high firing rates, and as a consequence, also across trials, leading to an artificial decrease. The calculation starts by extracting mean and variance of the spike counts for each combination of units and conditions (here, the 3 set sizes). We adopted sliding windows of 500 ms with a time step of 50 ms. The total numbers of points/combinations were 1254, 699, and 919 for hippocampus, amygdala, and entorhinal cortex, respectively. The greatest common distribution of mean spike counts across all the time windows was extracted (in practice, a histogram with bin size of 0.5 was employed). For each window, we discarded points randomly such that the common distribution was matched and, eventually, FF was determined as the slope of the line regressing variance over the mean of the remaining points. For the linear regression, the intercept was constrained to zero, and each point was weighted by the (inverse of 0.01 plus) standard error of the variance. Due to the multiple mean-matching possibilities, the procedure was repeated 50 times and the average FF with 95$\%$ confidence intervals (CIs) was reported. When comparing the set sizes directly (4 *vs* 6–8), we ensured that the same number of trials in each condition was utilized for computing the mean and variance of the spike counts. This was achieved by undersampling the condition with the highest number of trials. A different undersampling was performed for each combination of neuron and time step.

### Decoding analysis

Decoding analysis was performed on the neuronal pseudo-population obtained by collecting units across all of the sessions and patients. Spike counts were computed per unit in nonoverlapping windows of 250 ms within the 3-s maintenance period, thus yielding 12 points per trial. A $z$ score normalization was then applied to each of the neuronal series without any smoothing. The order and number of each grouping variable, e.g. set size, differ between each session, thus preventing an alignment of the trials of different sessions based solely on temporal succession. To this end, we describe in the following a bootstrap procedure adopted to calculate the decoding accuracies (Meyers et al. 2008). First, in each session, we identified the minimum between the number of trials belonging to each of the classes. If this number was lower than 5, that session was discarded. The minimum of these values across all of the sessions, $n_T$, was taken as the number of trials to be sampled per class within each session. Following this criterion, the classes’ instances were thus forcefully balanced. Second, after sampling the trials, the resulting data sets from each session were concatenated together creating the final pseudo-population activity matrix. The number of data points is equal to 12 $\times $ 2 $\times\,n_T$ given that we perform only binary classifications ($n_T$ = 9, set sizes 4 and 6–8; $n_T$ = 6, correct and wrong responses). At last, we train and test a linear support vector machine (SVM) following a 10-fold cross-validation scheme (SVC in Python package *scikit-learn*). The mean accuracy score across the 10 test sets was computed. The entire procedure was repeated 50 times, sampling thus different alignment configurations between trials of different sessions. To generate a null distribution of accuracy scores, we shuffled the class labels 500 times within each bootstrap cycle and repeated the procedure described above. One-tailed $P$-values were extracted and the median of these 50 quantities was taken as the summary value.

The total numbers of units for hippocampus, amygdala, and entorhinal cortex utilized when discriminating the set size were 418, 233, and 308, respectively; when classifying the response correctness, the numbers of units were correspondingly 103, 66, and 58. A large part of the decoding analysis is performed on subsets of these units defined by the $LvR$ values. The neuronal pseudo-population is split into n-tiles according to the mean $LvR$ value computed over the trials composing each particular bootstrap cycle. This implies that the same unit in distinct cycles can be assigned to different n-tiles.

To quantify the variation in accuracy when removing an n-tile with respect to the full population, we computed within each bootstrap cycle a d-prime or sensitivity index as follows:

(3) \begin{align*}& d^{\prime}=\frac{\mu_{Full} - \mu_{Red}}{\sqrt{0.5\cdot\left(\sigma^2_{Full} + \sigma^2_{Red}\right)}}, \end{align*}

where the subscripts indicate the full or the reduced neuronal population, and $\mu $ and $\sigma ^2$ are, respectively, the mean and variance of the accuracy scores across the 10 test sets. We ensured that the classification procedures of the 2 populations were performed on the same sample of trials. Eventually, we computed mean and SEM of the $d^{\prime}$ indexes and performed a $t$ test with zero as null hypothesis for the sample mean.

### Statistical analysis

All statistical tests were two-tailed unless stated otherwise. The utilization of nonparametric tests (Kruskal–Wallis, Wilcoxon rank sum, Wilcoxon signed rank tests) over parametric ones (ANOVA, $t$ tests) in one-way comparisons was decided upon significance of at least one Shapiro–Wilk test of normality on the samples involved ($P$ < 0.05). Post-hoc, pairwise tests were adjusted for multiple comparison with the Benjamini–Hochberg (BH) procedure. This multiple comparison adjustment is also the one applied in general where noted.

### Code and software accessibility

The analyses were carried out with R and Python packages available online. Customized code and scripts supporting the current study are available on https://gitlab.com/CaflischLab.

## Results

**Fig. 1:**
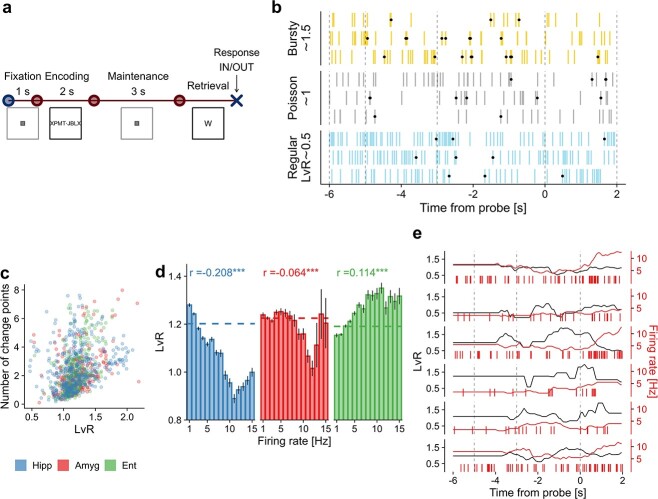
Memory task and firing irregularity of single neurons. (a) A modified Sternberg WM task was performed by epileptic patients. In each trial, a set of 4, 6, or 8 letters was displayed on a screen during the encoding period (2 s). A delay period followed (3 s; maintenance) and, after that, a probe letter appeared on the screen (retrieval). The subject then had to indicate whether the letter belonged to the set. (b) Examples of different firing patterns. These 3 stylized patterns derive from $LvR$ values distributed around 0.5, 1, and 1.5 for, respectively, regular, random (Poisson), and bursty dynamics. Dots indicate CPs. (c) Scatter plot between $LvR$ and the number of CPs. The 2 quantities are computed for each unit by averaging over single trials. (d) Relationship between burstiness and firing rates. Firing rates computed for each neuron (418, 233, and 306 units for hippocampus, amygdala, and entorhinal cortex, respectively) were grouped into 1-Hz bins. Mean $LvR$ values with SEM are shown for each bin. Dashed lines indicate the average $LvR$ values. Pearson correlation values between $LvR$ and firing rates computed over the whole neuronal population are shown on top. (e) Examples of time series of $LvR$ and firing rate values. Both of the quantities are calculated on a sliding window of 2-s with 50-ms steps. Windows are right-aligned to the time index (therefore, the curves are flat for the first 2 s). Burstiness levels for the population of maintenance and probe neurons (Boran et al. 2019) and trial-to-trial variability of the $LvR$ values are examined in Fig. S1.

The experiment is presented in Section Experimental design and  recordings (see also Fig. 1a and the description in the published data set (Boran et al. 2020)). In the following, we will use also the term “neuron” to indicate a unit.

We start by investigating the firing irregularities, or burstiness, of the single units by using a metric that relies on the interspike intervals (ISIs). We adopt a coefficient of local variation while correcting for refractoriness defined as $LvR$ (Shinomoto et al. 2003; 2009) (see Section Firing irregularities for details). The $LvR$ metric takes into account the local variations of consecutive ISIs, and its value characterizes the spike train dynamic as regular (LvR <1), random (Poisson-distributed, $\sim $1), or bursty (>1), as depicted in Fig. 1b.

In order to assess the validity of this metric, we used another proxy measure for quantifying irregularities in the spike trains, namely, the number of change points (CP). CPs are designed to locate and indicate sudden variations in firing rates. We hypothesized that a higher count of CPs will be observed for more irregular, burstier dynamics (see Fig. 1b as an example, see Section Firing irregularities for details). The number of CPs consistently exhibits significant correlations with $LvR$ in all of the anatomical areas ($r=0.20,\:t(17626)=26.5$; $r=0.24,\:t(9747)=24.8$; $r=0.26,\:t(13102)=30.5$ for hippocampus, amygdala, and entorhinal cortex, respectively; all $P$-values < $10^{-10}$, Student’s $t$ test) (Fig. 1c). Notably, the CP count does not fulfill our requirement of being independent of firing rates (correlations of $r\,=\,0.38,\,0.46,\,0.36$, respectively, as before; all $P$-values < 0.001), thus highlighting that the $LvR$ metric is a suitable approach to the nontrivial task of unveiling results that depend specifically on actual firing (ir)regularities rather than just rates.

###  

#### Irregularities show a nontrivial relationship with firing rates

By construction, $LvR$ is invariant to gradual firing rate fluctuations along time series, and, importantly, it does not depend on differences in spike counts between units (Shinomoto et al. 2003). In the following, we test the hypothesis that $LvR$ and firing rates have no trivial interdependence explicitly and in more depth. This is crucial for the remainder of the analysis as we desire to obtain information that is complementary to that provided by the firing rates for describing the spike sequences. We start in Fig. 1d by showing the average $LvR$ values for single bins of firing rate (1 Hz). In all of the 3 anatomical areas, the $LvR$ trend for increasing firing rates is neither linear nor strictly monotonic. Moreover, although some strong correlations appear in specific intervals of firing rates, e.g. in the hippocampus from 1 to 10 Hz, these dependencies are not generally replicated in the other anatomical areas. The diverse relationships between burstiness and firing rate can also be observed in the global Pearson correlation values, which assume different values and signs in hippocampus ($r=-0.21;\:t(18377)=-28.8,\:P\,<\,10^{-10}$, Student’s $t$ test), amygdala ($r=-0.06;\:t(10148)=-6.4,\:P\,<\,10^{-10}$), and entorhinal cortex ($r=0.11;\:t(13504)=13.3,\:P\,<\,10^{-10}$). Importantly, $LvR$ displays nontrivial behaviour also locally in time, meaning that sudden changes in firing rates do not always trigger the same $LvR$ variations, as highlighted by the examples in Fig. 1e.

#### Burstier patterns are present in all of the anatomical areas although they appear to be volatile across trials

Having established a substantial independence from firing rate values, we move now on to the specific measurements on $LvR$ alone in this data set. On average, we observe a prevalence of irregular patterns (LvR = 1.19 [0.89,1.42], 1.22 [0.93,1.48], 1.19 [0.95,1.42], for hippocampus, amygdala, and entorhinal cortex, respectively; mean and interquartile range (IQR) over all of the trial$\times $unit combinations). For the sake of completeness, the maintenance and probe neurons introduced in Boran et al. (2019) were also inspected. These were identified as those with a higher spike count during the maintenance or probe period, respectively, when compared with the fixation period. As shown in Fig. S1a, maintenance neurons display significantly lower $LvR$ values with respect to the other units in the entorhinal cortex ($P$ = $0.026$, Wilcoxon rank sum test). For probe neurons, the difference depends also on the anatomical area: in the hippocampus, they are associated with lower $LvR$ values ($P$ = $0.040$) while in the amygdala with higher ones ($P$ = $0.024$). Evidently, it is difficult to draw conclusions on a definite relationship between irregularities and sustained firing rates as those shown by maintenance and probe neurons.

Next, we assessed whether the firing behaviour remains stable within a recording session by investigating the trial-to-trial variability of $LvR$ of individual units, see Fig. S1b. This is examined by simply computing the mean and standard deviation of the $LvR$ values separately for each unit across trials. The correlation values between standard deviation and average exhibit positive to (weakly) negative values when examining in this order hippocampus ($r$=0.573, $P$ < $10^{-10}$), amygdala (0.225, $P$ = 0.0005), and entorhinal cortex (-0.126, $P$ = 0.027). Thus, in both hippocampus and amygdala, a neuron displaying burstier patterns will tend to do so inconsistently across trials, whereas the opposite (to a weak extent) is observed in the entorhinal cortex.

#### $LvR$ values are modulated selectively but weakly by trial parameters in the different brain regions

**Fig. 2:**
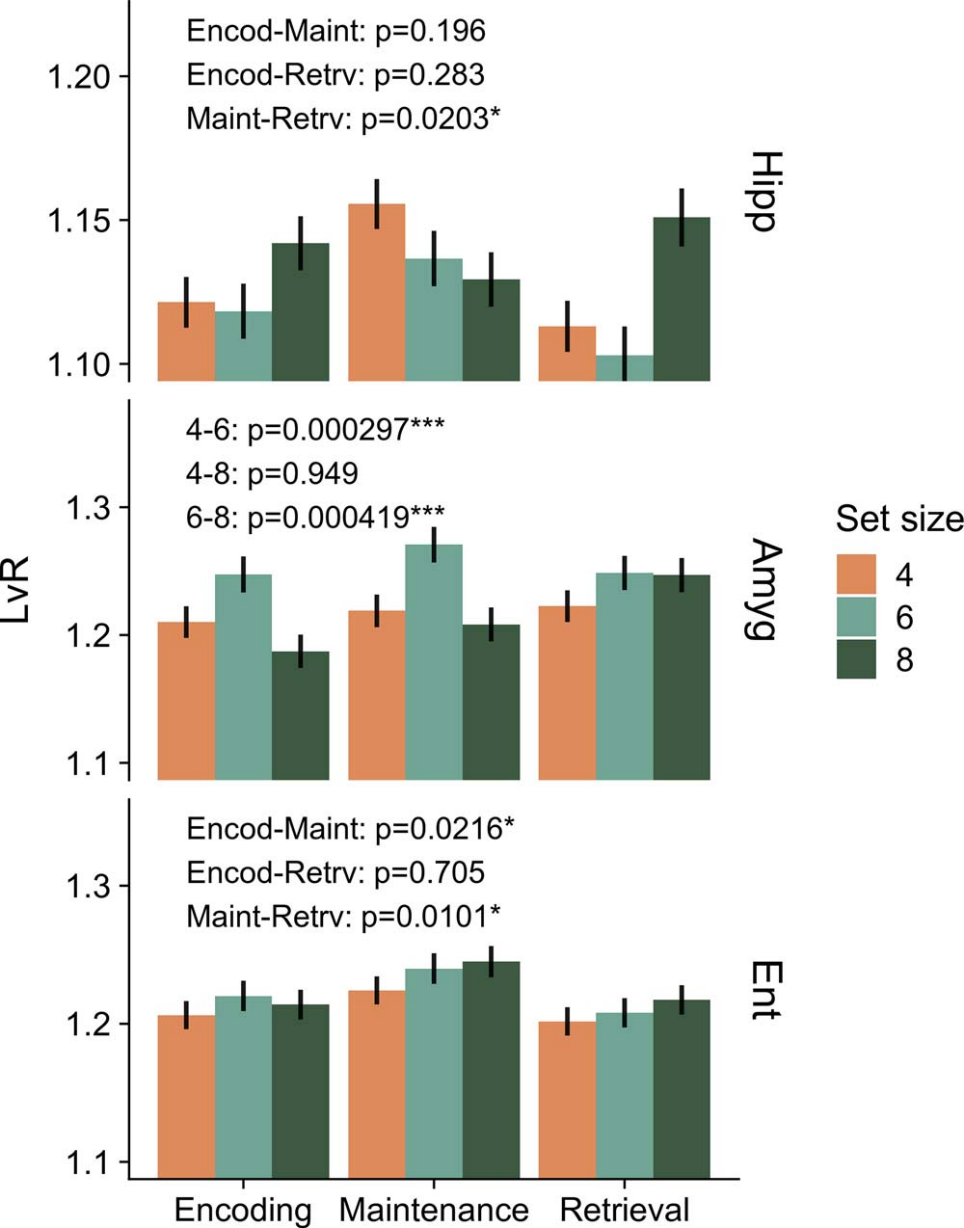
$LvR$ values resolved by combination of anatomical area, set size, and trial period. $LvR$ values were calculated on 2-s windows centered on the respective trial periods. A two-way ANOVA test with trial period and set size as factors was performed beforehand within each anatomical area. Only factors associated with significant main effects were examined further (interaction effects were included in the model but they did not reach significance). In particular, we show here the results ($P$-values) of post-hoc pairwise $t$ tests (*$P$ <0.05, **<0.01,***<0.001, BH-corrected).

Next, we inspect the relationship between burstiness and the behavioural and trial variables. In preparation, we created a three-way ANOVA model between $LvR$ and three factors, namely, set size, correct/wrong response, and trial period. The $LvR$ was computed for each unit on 2-s windows which were centered in the respective trial periods (encoding, maintenance, retrieval; fixation was excluded due to its short duration). Since we required that each session should contain at least 5 trials with wrong responses (out of $\sim $50), we collected data from only 7 sessions. The analysis delivered no significant results for the main effects, except for the trial period in the hippocampus, which is investigated later. We removed the correct/wrong response factor to enable us to perform the ANOVA test with a larger number of sessions (26, using only trials with correct responses). Trial period appeared to modulate the $LvR$ response in the hippocampus ($F_{2,31502}$ = 3.77, $P$ = 0.023, $\omega ^2$ = 0.0002) and the entorhinal cortex ($F_{2,23340}$ = 5.11, $P$ = 0.006, $\omega ^2$ = 0.0004), whereas set size affected only the amygdala ($F_{2,16868}$ = 9.42, $P$ = $8\cdot 10^{-5}$, $\omega ^2$ = 0.001). It must be noted, however, that the effect sizes ($\omega ^2$) are very small. Thus, it is likely that other factors, behavioral or nonbehavioral, are required to explain the total variance in $LvR$. In Fig. 2, we show the results of the pairwise tests corresponding to those significant main effects. During maintenance periods, higher $LvR$ values are observed in the entorhinal cortex and hippocampus, although the comparison was significant only with respect to the retrieval period for the latter. In the amygdala, on the other hand, we observe significantly higher $LvR$ values for set size 6 when compared with both 4 and 8.

### Irregularities in unit combinations and their relationship with response times

**Fig. 3:**
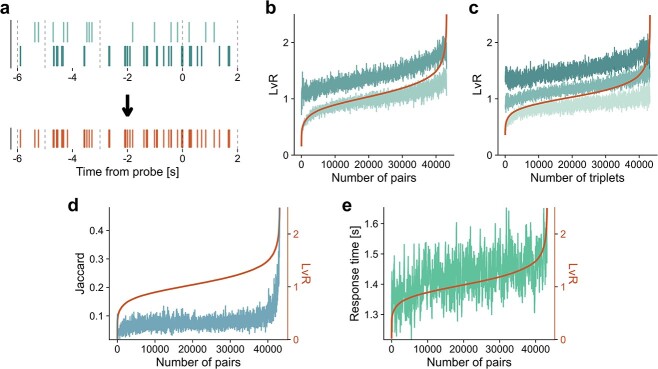
Combinations of pairs or triplets of spike trains. (a) Spike trains of two units recorded simultaneously (top) are combined into a new spike series. The dashed, vertical lines highlight the time structure of a trial (fixation, encoding, maintenance, retrieval). (b) $LvR$ values resulting from the combination of 2 spike trains are sorted (red line) and shown along with those of their individual components (green traces). See Fig. S2 for an alternative representation of these results where the single-unit values are sorted. (c) Same as in (b) but showing $LvR$ values deriving from the combinations of three spike trains. (d) Combined $LvR$ values and correlations between spike trains. Jaccard values were computed between pairs of binarized spike trains (50 ms bin; blue line). These values are reordered with respect to the combined $LvR$ value obtained from the corresponding unit pair (red line). (e) Combined $LvR$ values and response times. Similarly to (d), the sorted sequence of paired $LvR$ values (red line) is plotted but here along with the (reordered) response time of the related trials (green). In panels (b)–(e), data from all of the sessions and anatomical areas are used; not all of the combinations are plotted but a subsample that preserve the relative contribution of each session in terms of recorded units. A moving average filter with a window of $10$ and $60$ points is applied in panels (b)–(d) and panel (e), respectively, for visualization reasons ($\sim $40 000 points are shown).

Irregularities can also occur and be monitored at the ensemble rather than the single-unit level; for this reason, we extended our analysis to include spike patterns resulting from pairs and triplets of units. In practice, for the latter, we combined the spike trains of 2 or 3 simultaneously recorded units and computed the $LvR$ measure on the joint time series (see Fig. 3a). The rationale behind this procedure is that among the characterizations of a ‘persistent’ activity, the spike train of a single unit can appear sparse and irregular. However, simultaneously recorded units can fire asynchronously and together fill the activity gaps during the delay period, showing ultimately persistent and, potentially, more regular firing patterns as a whole (Lundqvist et al. 2018). On the other hand, irregular patterns can be ultimately reinforced by the combination of concomitant bursty patterns. It is thus interesting to investigate whether genuine coordination between units is present and to what extent they carry information on the trial variables.

In Fig. 3b and c, we show the resultant burstiness values (red curves) combining 2 and 3 individual spike trains, respectively (green curves). In this and in following analyses, only random subsamples of all possible combinations are utilized (see Section Time-resolved analysis of irregularities and trial variables). The relation between the individual components’ $LvR$ and the combined ones is, not surprisingly, monotonic on average (the curves shown are smoothed). The combined value remains closer to the smallest component in $LvR$ (lighter hue) rather than to the highest one (darker hue) for a large fraction of the total number of combinations (around $\sim $80%) and $LvR$ spectrum (up to $\sim $1.2 for the combined value). The combined $LvR$ thus seems to depend considerably on correlations between units in the opposite cases of strong and weak values. This is shown in Fig. 3d, where the Jaccard similarity measured derived from 50-ms bins has been adopted (not be confused with the use of the Jaccard measure described in Section Experimental design and recordings). The lowest $LvR$ values, i.e. the more regular trains, tend to be formed by uncorrelated units; on the other hand, there is a steep surge in burstiness for highly correlated neurons. This can be understood from the fact that closely coordinated spikes create smaller ISIs in the joint spike train due to overlapping bursts. This contrasts with two uncorrelated, bursty neurons, where the overlapped train is unlikely to see a strong increase in short ISIs because the burst periods do not align.

#### Burstiness shows correlations with response times independently of firing rates and with larger effect sizes for unit combinations

**Fig. 4:**
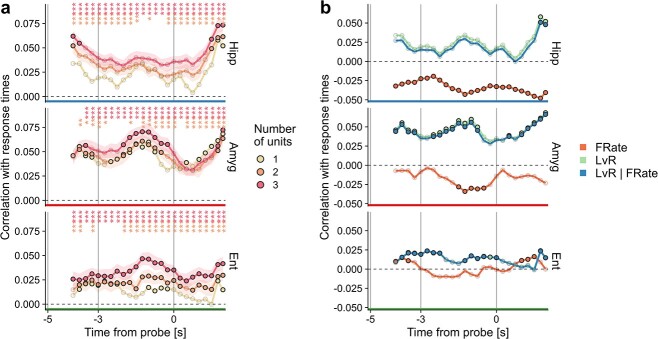
Relation between time-resolved $LvR$ values and response times. (a) Pearson correlation between $LvR$ and response time. $LvR$ and correlation values are reported for right-aligned sliding windows of 2 s with 250 ms step ($n$=11 283, 6192, and 8544 points for hippocampus, amygdala, and entorhinal cortex, respectively). Error bars on joint spike trains of 2 and 3 units (95% CI) derive from the repeated subsampling of unit combinations (100 times; similarly to Fig. 3b–e, not all of the possible combinations are utilized, see Section Time-resolved analysis of irregularities and trial  variables for details). The opacity of symbols denotes significance of the respective correlation value when compared with a null distribution ($P$-value < 0.05; permutation test). Significant differences between the combined values and the single-unit ones are indicated on top ($t$ test, *$P <$0.05, **<0.01,***<0.001). The gray vertical lines delineate the encoding, maintenance, and retrieval periods. In Figs. S3 and S4, a similar approach is used for investigating the relationship between *LvR* and either set size or correct responses. (b) Contribution of firing rates. The same procedure as in panel (a) was adopted for computing the correlations between firing rates and response times. Partial correlations between $LvR$ and response times, while controlling for firing rates, are included (‘LvR | FRate’) and often overlap with the $LvR$ data. Only single-unit calculations are shown. See Section Time-resolved analysis of irregularities and trial variables for details.

An interesting behavioural variable yet to be analyzed here is the response time of the patients, which provides a proxy measure for the level of attention as well as for the perceived complexity of the task. In the following, we looked for possible relationships with burstiness values. When simply ordered with respect to the $LvR$ values, the response times reveal a clear increasing trend (Fig. 3e). We were curious if and how this effect varies along the time axis of the trials and if its magnitude depends on the coordination between units. To this end, we computed Pearson correlations between the response times and time-resolved $LvR$ values of both single and combinations of units computed on sliding windows of 2 s, see Fig. 4a. The significance of each value is assessed with a permutation test respecting the trial structure (transparency levels; $P<$0.05, BH-adjusted across the anatomical areas). The set of combined $LvR$ values was subsampled in each session in order to match the number of units used for the single $LvR$ results. In this way, we guarantee a fair comparison between statistics for the different levels of combinations. More details are in Section Time-resolved analysis of irregularities and trial variables.

For all of the anatomical areas, higher correlation values are observed almost everywhere for the combinations of 3 units (Fig. 4a). The differences between paired and single-unit $LvR$ values are less pronounced in comparison. Hippocampus and amygdala show a marked modulation with respect to the specific time window. Both of the 2 regions reach significant Pearson correlations in encoding/fixation and in the last phase of each trial. In addition, this holds for the amygdala also for a large fraction of the maintenance period. In the entorhinal cortex, most time windows show weak but significant correlation but only if we consider combinations of 2 or 3 units. A similar time-resolved analysis was performed for the categorical variables (set size and correct response, see Fig. S3); however, we could not observe clear or significant relationships of the $LvR$ values with these 2 variables for either single or combinations of units.

Could the correlations with response times be explained in terms of firing rates? We repeated the analysis using firing rates of single units instead of $LvR$ and show the results in Fig. 4b. Generally, firing rates tend to anticorrelate with the response times with lower absolute magnitudes, except for the hippocampus where the significance threshold is met everywhere. In spite of the fact that the profiles approximately mirror those of $LvR$, the correlations between $LvR$ and response times seem to be unaffected by firing rate values, as shown by a partial correlation analysis (blue line, Fig. 4b).

### From single-unit to population analyses in the context of trial-to-trial variability

**Fig. 5:**
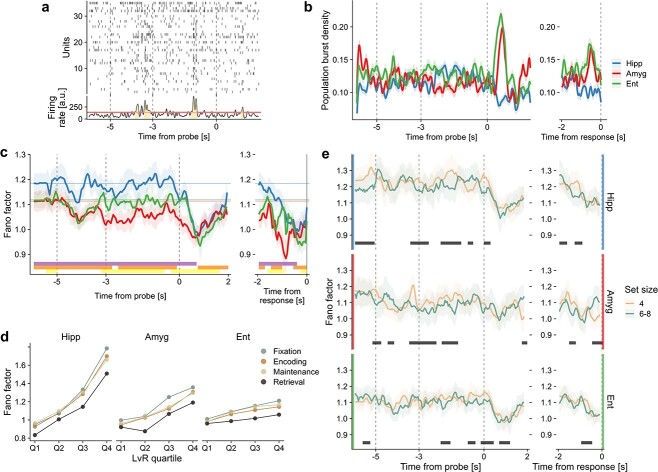
Population burst and trial-to-trial variability. (a) Example of population burst events. Spike raster plot of an example trial (top) and the global firing rate (bottom) are shown. Burst events are defined as events longer than 100 ms where the collective firing rate exceeds a data-derived threshold. See Section Population bursts for details. (b) Population burst density per anatomical area. The densities were calculated at each time bin (10 ms) as the fraction of trials where a burst event was ongoing. Bootstrapping at the trial level (1000 times) was used to arrive at the 95% CIs that are shown. The numbers of trials analyzed were 993, 809, and 806 for hippocampus, amygdala, and entorhinal cortex, respectively. Time traces aligned with respect to the response time are shown on the right. In this case, trials with response times higher than 2 s were discarded ($\sim $110 trials). In Fig. S5, we show the burst density profile computed excluding the probe neurons. Further measurements regarding the bursts’ properties and their relationship to the different sizes are provided in Figs. S6–S8. (c) Fano factor per MTL region. Calculations were performed on right-aligned sliding windows of 500 ms with a 50-ms time step using a mean-matching procedure for controlling variability in firing rates (see Section Fano factor for details). Mean and 95% CIs are shown. Horizontal lines represent the average value of FF during the fixation period. Vertical gray lines delineate trial periods. On the right inset panel, spike trains from the different trials are instead aligned to the response time before calculating the FF. Results of a cluster-based nonparametric permutation test are shown on the bottom (*P* < 0.05; violet: ‘Hipp’ *vs* ‘Amyg’; orange: ‘Hipp’ *vs* ‘Ent’; yellow: ‘Amyg’ *vs* ‘Ent’). (d) The same FF calculation as in c) was repeated by splitting the neuronal population into 4 quartiles on the basis of their mean $LvR$ values. The average FF within each trial period is reported. The results of ANOVA and pairwise tests between the different combinations of quartiles and trial periods are reported in the main text. (e) Same as (c) but distinguishing set sizes (4 *vs* 6–8) for each anatomical part. Results of a cluster-based nonparametric permutation test are shown on the bottom ($P$ < 0.05). A moving average filter of 4 points (200 ms) was applied in panels (c) and (e) to improve readability.

While the behavior of individual units is informative, it is almost certainly primarily symptomatic of higher order processes taking place at the level of ensembles of neurons (compare Fig. 4). Thus, we next proceeded to examine irregular patterns at the population level. In particular, we characterized population burst events and their distributions. These events were investigated for two main reasons: first, they represent episodes which can be relevant for the underlying memory processes (Vaz et al. 2020); second, they constitute a coordinated activity between units and, thus, it might be interesting to check whether regular or irregular activities mediate this coordination. More in detail, a population burst is defined as a period where the collective activity is sustained and persists above a specific threshold computed by pooling all of the trial data, see Fig. 5a and Section Population bursts for details. Distinguishing between the bursty activity of single units (quantified by $LvR$) and population bursts is important as the latter are not a generalization of the approach taken in Fig. 3. For ease of reading, in this section, we will often refer to population bursts simply as “bursts”.

#### Population burst activity is sharply modulated after probe presentation

First, we analyzed the population burst density in a time-resolved manner, and this is shown in Fig. 5b, which plots the fraction of trials that display an ongoing burst event. Focusing on the hippocampus, there is a slight increase in the first 2 s of the maintenance period followed by a quick drop after the appearance of the probe letter. This behaviour is reversed for the amygdala and entorhinal cortex where an abrupt increase of burst activity within 1 s of probe presentation is apparent. This enhancement is likely to be triggered by the probe onset itself rather than being related to the initiation of movement or to the effective memory retrieval process. This is evident from the fact that the effective burst density is reduced if the time series are aligned to the effective response time (inset to the right), although with smaller effect for the amygdala. Average burst numbers and lengths confirm this picture (Fig. S6a and b) but the absolute differences across regions and trial periods are small. Can this enhanced activity be explained by the probe neurons investigated in Boran et al. (2019)? In Fig. S5, we measured the population bursts again but excluding the probe neurons from the analysis. When aligned to the probe presentation (left panels), the peak is indeed reduced in the entorhinal cortex (although still prominent), but it remains unaltered in the amygdala. On the other hand, a reduction in this area does become visible when data are aligned to the response times. Given that, we suspect that the population burst activity stemming from the probe onset *per se* is likely to be evenly distributed across the population. In contrast, the signal associated with the effective memory retrieval (if any) is likely to be captured by a limited set of neurons, such as the one represented by the probe neurons.

#### Properties of population bursts fail to inform on workload

Given the negative results in Fig. S3 for $LvR$, we wondered whether population bursts show clear correlations with set size. Somewhat surprisingly, Fig. S7 shows that partitioning the data into set size 4 *vs* 6–8 creates results that are generally indistinguishable from each other, posing the question whether the frequency and length of population bursts is more tied to anatomical features than to representation. Thus, we also considered burst composition as a feature. However, in Fig. S6c, the sparsity of bursts (how many units contribute to a burst) is revealed to offer little contrast across regions and periods remaining at close to 50%, and consistent with this, we could not identify significant differences in burst compositions for different set sizes relative to a reference, see Fig. S8. The comparison of averaged single-unit $LvR$ values and the weighted average across units while bursting in a population event (Fig. S6d) offers some unexpected and significant trends but, again, primarily with anatomical region and not with trial epoch.

#### Trial-to-trial variability is lowest during retrieval

A suitable measure of the dispersion of our results across trials is not only an important adjudicator of statistical relevance but also an indicator of the presence of heterogeneous biological mechanisms. Here, we turn to a Fano factor (FF) analysis of the variability across trials to answer questions such as, for example, how consistent are changes in population burst activity across trial epochs? We start by examining the average FF values for the 3 anatomical areas along the trial time (Fig. 5c). The hippocampus displays variability values that are generally higher compared with the other 2 areas (cluster-based permutation test, $P$ < 0.05, Maris and Oostenveld (2007)). Amygdala and entorhinal cortex assume distinct values in particular during maintenance, with the amygdala showing more stable firing patterns across trials. Generally, all of the areas offer a similar picture during the presentation of any stimulus; specifically, we recognize a drop in FF after the first second of the encoding period ($ ca.$-4 s) as well as of the retrieval period ($ ca.$1 s). For the latter, the result appears consistent with the population burst dynamics of Fig. 5b where a sustained activity of amygdala and entorhinal cortex emerged with similar timing relative to the probe presentation. On the other hand, in Fig. 5c, also the hippocampus shows a reduced variability, suggesting a consistent decrease, rather than increase, in burst activity across trials. The inset (right panels) allows the interesting observation that, unlike hippocampus and entorhinal cortex, the FF of the amygdala is not minimal just before the time of response but rather $\sim $1 s earlier. Moreover, it reaches lower FF than those shown when aligned to probe presentation. Activity in the amygdala thus seems to be more directly linked to the response times (1 s in advance); this might be indicative of the fact that the amygdala is more involved either in the preparation of movement or in memory retrieval than the other anatomical regions.

#### Fano factor increases with burstiness values and helps in discriminating the workloads

What is the relationship of FF with the irregularities of the spike sequences? The answer to this question is not a trivial correspondence. For example, for spike trains exhibiting regular patterns, the firing rates could either vary (high FF) or remain constant (low FF) across trials. Similarly, bursty sequences could display either asynchronous patterns (high FF) or coordinated activity during specific epochs (low FF) (Constantinidis et al. 2018). If no specific time-locked activity is present, we do expect higher FF for higher $LvR$ values. In Fig. 5d, we split the units into 4 quartiles based on their mean $LvR$ values and recomputed the FFs. The average values within each trial period are shown. A two-way ANOVA with trial periods and $LvR$ quartiles was performed beforehand and showed significant main effects for both of the factors in all of the anatomical areas ($F_{3,481}\,=\,[153.2, 4240.1],\,[110.0, 964.8],\,[94.5, 298.2]$ and $\omega ^2\,=\,[0.03, 0.93],\,[0.09, 0.78],\,[0.17, 0.54]$ for hippocampus, amygdala, and entorhinal cortex, respectively; all $P$ < $10^{-10}$. For the $F$- and $\omega ^2$-values, the 2 numbers in the parentheses refer to the main effects of trial period and $LvR$ quartile, respectively. The higher the burstiness (i.e. the quartile), the larger is the trial variability in virtually all of the anatomical areas and trial periods (Wilcoxon signed rank tests, all $P$ < $10^{-7}$). Hippocampal units span a much larger spectrum of FF values when compared with units in the amygdala and the entorhinal cortex, and all of the quartiles tend to preserve roughly the same differences between the trial periods, with the retrieval epoch exhibiting the lowest FF values in all combinations of anatomical areas and quartiles (Wilcoxon rank sum tests, all $P$ < $0.01$).

To conclude this analysis, we investigated whether the memory workload modulates the trial-to-trial variability. When discriminating set sizes (Fig. 5e), we observe significant differences in FF values in all of the trial periods, including fixation. We cannot exclude the caveat that this difference could arise from the fact that the trial conditions were not always sampled independently (see Section Experimental design and recordings). Set size 4 yields significantly higher dispersion than 6–8 for much of the maintenance period in hippocampus and amygdala and also during retrieval when aligned to response time (right panels). This suggests that higher workloads require more stable activity for both memory phases although this seems to loose stringency at the end of maintenance, possibly due to difficulties in retaining the stimulus content. We observe also that the anticipation of minimal FF with respect to the response time of the amygdala is primarily the result of higher workloads (6 and 8). Given that, we conjecture that this signal involves processes related to mnemonic functions rather than movement ones. During encoding, however, it is not possible to associate the (weaker) FF decrease on the first stimuli presentation with a specific workload (Fig. 5e, main panels).

### Decoding analysis

In the final part of our analysis, we asked ourselves whether the decoding capabilities of the population activity observed in Boran et al. (2019) depend on their firing irregularities. In particular, is the decoding of the workload dependent on the $LvR$ values of the underlying populations? In order to assess the importance of the firing behaviour, we split again the neuronal pseudo-population, obtained by pooling all of the sessions, into 4 quartiles defined by the average $LvR$ value of the units (compare Fig. 5d). For each of these subpopulations, a linear decoder was trained and tested following a cross-validation procedure. In order to avoid confusion with the analysis of Fig. S3a, we remark that the decoding is performed in an N-dimensional space, where N represents the number of neurons, using the (normalized) firing rates. Overall, we follow the analysis performed in Boran et al. (2019) but with two main differences: we use here a decoding scheme based on bootstrap for the alignment of trials belonging to different sessions, and we distinguish the different units according to their burstiness typology (see Section Decoding analysis for more details).

#### Neural populations showing non-Poisson firing predict the memory workload more accurately

**Fig. 6:**
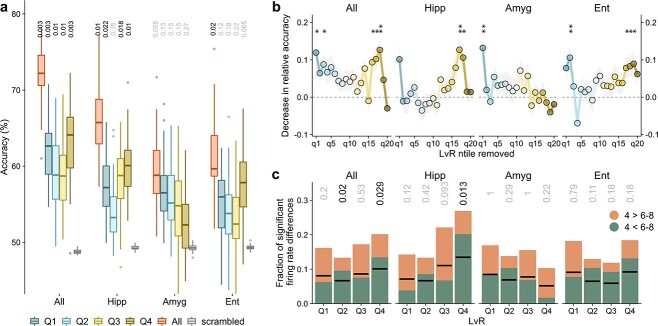
Decoding of trial variables during the maintenance period for different firing patterns. (a) Decoding performances of set size (left) during the maintenance period are evaluated on neuronal subpopulations split into 4 quartiles according to their $LvR$ measure (blue to yellow boxplots). Decoding accuracies for the whole set of units are shown in red along with chance values obtained by shuffling the trial variable labels (grey). The boxplots represent the distribution of 50 accuracy scores obtained by a bootstrap process aimed at sampling different alignment configurations between trials of different sessions. Within each bootstrap cycle, a statistical test is performed using the chance level values obtained by scrambling the labels (500 times). The 50 resultant $P$-values are summarized in the median value shown on top of each boxplot (BH-corrected within each anatomical area). We employed a balanced linear SVM model trained and tested with a 10-fold cross validation procedure. See Section Decoding analysis for more details. (b) Contributions of $LvR$ subpopulations to the decoding performances. The higher the loss in accuracy ($y$-axis), the larger is the contribution of the specific $LvR$ n-tile to the decoding ($x$-axis). Similar to a), neuronal populations are split into 20 n-tiles according to their $LvR$ values, and, in turn, each n-tile is removed and the decoding procedure repeated as above. Here, we capture the normalized decrease in accuracy performances with respect to the full population scores (see Section Population bursts). Mean and SEM of the indexes computed across the bootstrap runs are shown. Results of a $t$ test with zero as expected sample mean are reported on top ($df$=49, *$P<$0.05, **<0.01, ***<0.001; BH-corrected across the different n-tiles). In Fig. S9, the same analysis of panels (a) and (b) is presented for the decoding of correct/wrong responses. (c) Differences of single-unit firing rates between different set sizes. For each unit, a $t$ test between the firing rates of trials with set sizes 4 and 6–8 is performed (data points are the same as those utilized for panels (a) and (b)). The fraction of units yielding a significant test is reported while distinguishing also the sign of the $t$ statistic. Black segments are positioned at half height of the respective columns to guide the eye. Binomial tests on the number of occurrences of significant positive (or negative) $t$ statistics were performed ($P$–values shown on top).

We show the results of the analysis for the maintenance period in Fig. 6a. We observe that encoding of set size generally benefits from all of the anatomical areas and from the presence of all of the neurons irrespective of their burstiness type. However, when grouping the neuronal populations by their $LvR$ values, some differences arise between the anatomical areas. Units in the hippocampus show significant decoding accuracies for all of the quartiles except for the second one (corresponding to $LvR$ values between 1.03 and 1.16), with the most irregular neurons performing best. A decreasing trend in performance is observed for the amygdala, whereas units in the entorhinal cortex approach the significance threshold only on the last quartile ($LvR$ > 1.30, $P=0.065$) encompassing the most irregular neurons.

To corroborate these results, we explored a complementary approach to investigate at finer resolution how firing characteristics affect the decoding capabilities. Rather than splitting the whole population into nonoverlapping subpopulations, we remove in turn a small set of neurons equal to 1/20 of the total number characterized. This small set, in the same spirit as before, corresponds to a specific $LvR$ n-tile. The resultant decoding accuracies are compared with the full population ones, which allows a fairer comparison between the 2 measures in terms of dimensionality of the decoding space (differing by only 1/20 and not by 3/4 as before). In more detail, we compute sensitivity indexes between the accuracy scores of full and depleted populations while accounting for the variability between the cross-validation folds (see Section Decoding analysis). The results are shown in Fig. 6b. Qualitatively, the same patterns reported in panel (a) concerning the set size decoding emerge also at finer resolution. However, wider variations between contiguous n-tiles are resolved, especially close to boundary values. For example, in the hippocampus, the very last n-tiles do not contribute significantly, and generally, across all of the anatomical areas, neurons firing with regular patterns contribute only with the lowest $LvR$ values (first one or two n-tiles). Importantly, and this holds for all of the anatomical areas, the random (Poisson) patterns seem to carry no relevant information about the workload. We applied the same procedure of Fig. 6 for decoding correct and wrong responses from the population activity during maintenance (Fig. S9). Somewhat surprisingly, not a single set of units reported a significant accuracy.

#### Single-unit activity offers insights into mechanisms of workload decoding

In the final part of our analysis, we tried to shed light on the previous decoding results by investigating how the single-unit firing rates coded for workloads in the different $LvR$ quartiles. In Fig. 6c, we plot the fraction of single units which displayed significant differences in firing rates between the set sizes (4 *vs* 6-8 as for panel (a)). Qualitatively, the results across the quartiles follow the same trends as observed in Fig. 6a, hinting at the fact that much of the workload information is stored in single-unit activity. Given this, it is interesting to observe that for the most irregular and significant quartiles of Fig. 6a (that is, for “All” and “Hipp”), the number of units responding for large set sizes (6–8) is significantly higher than that favouring lower workloads (binomial test, Fig. 6c, top). We therefore conjecture that burstier units tend to possess a simpler code for the workloads, which is based on a coordinated increase in firing rates rather than on an asynchronous mixed activity across units.

## Discussion

The discharge patterns that are observed in the MTL during different memory phases are hypothesized to contain information about the memory items as well as shed light on the underlying neuronal dynamics that govern the memory processes. Differently from the approaches adopted in many prior works (Bausch et al. 2021; Boran et al. 2019; Derner et al. 2020; Kamiński and Rutishauser 2020; Kamiński et al. 2020; Kornblith et al. 2017) (see also Rutishauser et al. (2021)), we did not, except for controls following Boran et al. (2019), distinguish neurons based on their firing rate levels associated with external variables and observables, such as trial periods, task conditions, or memory items. Such an approach contains the implicit assumption that only a small fraction of neurons carry information about behaviour and stimuli. Instead, we kept our focus on the whole set of recorded neurons and analyzed how the (ir)regularity levels at different resolution stages, i.e. single-unit, unit combinations, and population level, appear to inform on the different trial variables. Furthermore, we moved away from a spike rate-centric approach and from an analysis driven by the external variables and instead focused on the internal activity (Buzsáki 2020). Such an analysis has higher exploratory power as it is able to assess also WM models that differ from the ones built on the hypothesis of persistent neuronal activity (Kamiński et al. 2017).

###  

#### Burstiness levels are indicative of response times and attention levels

Our analysis is based in large part on the quantification of the irregularities of spike trains through the $LvR$ metric, which has a nontrivial albeit weak interdependence with net firing rates (Shinomoto et al. 2003) (Fig. 1d and e). We found that higher $LvR$ values and, thus, burstier patterns tend to be associated with higher response times, with particular emphasis for the entorhinal cortex and the amygdala during the delay (maintenance) period. Irregularity patterns resulting from the combinations of multiple units (two and three) were generally more correlated with observed response times, suggesting that ensemble-level coordination between units can better encode behavioural responses. Moreover, since burstier patterns, which are associated with slower responses, are related to higher correlations between units (Fig. 3d), it would be interesting to explore whether they play a role in describing/controlling attention levels (as in Cohen and Maunsell (2009)). The fact that significant correlations are observed in the beginning of the encoding period, comprising also fixation, suggests that the attention level should be considered when interpreting the results obtained.

The relationship between burstiness and attention has been previously investigated, especially in nonhuman primate studies. For example, in the anterior cingulate and the lateral prefrontal cortex, an increase in the number of single-unit bursts was identified during different states of attention and associated with a functional synchronization between the 2 different brain areas (Womelsdorf et al. 2014). These results are seemingly at odds with ours: however, it must be considered that the definition of single-unit bursts adopted therein is highly dependent on the spike counts. Differently, a net distinction between firing rates and burstiness was highlighted in Anderson et al. (2013) (see also Xue et al. (2017)); the authors observed 2 concurrent attention-dependent phenomena in the V4 cortical area of macaques: an increase in firing rate and a reduction in burstiness. There, the assessment of burstiness relied on the distribution of ISIs, thus making their results more comparable to ours in terms of both the chosen metric and of the observed behavioral relationships (i.e. higher attention levels corresponds to lower burstiness).

Importantly, the relation between attention and WM is not yet clear, both on the behavioural level but especially in its realization in the neural substrates (Oberauer 2019; Shevlin 2020). Our results hint at the discharge patterns as a variable of interest to disentangle the aforementioned relationship. For example, specific memory activity might be mediated by spike counts of the maintenance neurons of Boran et al. (2019) or more generally through memory-selective cells (Rutishauser et al. 2021), while the nature of the discharge patterns might relate only to attention-related variables. Indeed, we did not find any relevant workload dependence of the $LvR$ values in the MTL regions, except for a puzzling preference in the amygdala of high burstiness values for the intermediate set size 6 (Fig. 2).

#### Persistent *vs* transient activity: potential integration of the two models

Considering the discussions around the competing theories underlying WM dynamics (mostly) in the prefrontal cortex (Constantinidis et al. 2018; Lundqvist et al. 2018; Masse et al. 2020), comparatively less research on this topic has been performed relying on data from the MTL areas analyzed here (Boran et al. 2019; Kamiński et al. 2020; Kornblith et al. 2017). The main discussion between the prevalent models postulating asynchronous, persistent activity against those which argue for a dominant role of transient (bursty) but coordinated (or also silent) activity is hampered by the definition of “persistent” activity. This is a term that is adopted widely in the literature but, as observed in Kamiński et al. (2017), it frequently remains ambiguous and lacks a sound quantitative definition. For example, it is not always clear which neural substrates and at which resolution this activity should be observed and defined as such, i.e. single neuron (more often), population level and local networks, or brain oscillation (Leavitt et al. 2017). In addition to that, it must be remarked that each brain area can show intrinsically diverse activity characteristics during the delay period; this activity, in turn, can depend also on the stimulus type and on the structure of the memory tasks (Christophel et al. 2017; Leavitt et al. 2017; Sreenivasan and D’Esposito 2019). Here, we choose to equate persistence with the regularity (or randomness) of the spike trains and the competing transient activity with the burstier patterns (high $LvR$). However, for the aforementioned reasons, it is important to always interpret this terminology with caution.

Our analysis shows that the successful retrieval of information in memory (correctness of the response) and the workload (set size) cannot be decoded with clarity from the spectrum of burstiness values of single units or combinations thereof (two or three) during the maintenance period (Figs. 2 and S3). Similarly, the frequency and properties of population bursts were not helpful toward this goal either (Fig. S7). On the other hand, when decoding the workload from the (pseudo-)population activity patterns, our results suggest that in the hippocampus and, to a lesser extent, in the entorhinal cortex, both neurons with regular and with bursty patterns concomitantly provide a significant contribution toward decoding performance with the latter showing slightly larger accuracies (Fig. 6a and b) when their respective contributions are isolated. Possibly, the burstier the neurons, the more linear is the relationship between firing rates and set size, favouring thus a better discrimination of workloads already at the single-unit level. This would be consistent with the data for the hippocampus in Fig. 6c where the quartile of highest burstiness is the only single-region one returning a significant result on the binomial test measuring preference for which set size corresponds to higher firing rates. In contrast to the other 2 regions, neurons in the amygdala tend to exhibit better performances the more regular their spiking activity is. Our results thus do not exclude the possibility of an integration of the two aforementioned competing models (transient and persistent) within the same brain system. However, as mentioned above, we cannot and should not rule out the possibility that one signal corresponds to the actual memory content, while the other encodes the level of general attention that can certainly be modulated by the memory load. This concurrent encoding based on single-unit (ir)regularity might be compatible with the multiplexing of information by single isolated spikes and rapid succession of those at the population level (Naud and Sprekeler 2018; Oswald et al. 2004).

In a recent work (Li et al. 2021), it was shown that computational models of WM implementing either burst-coding or elevated persistent activity could be distinguished by measurements of trial-to-trial variability. In particular, for the first class of models (“burst-coding”), the authors predicted an increased FF during the delay period, while the second class (‘elevated persistent’) displayed more stability across trials (lower FF). Our own results do not favor either model clearly. We did not in fact observe variations in FF between the fixation and the maintenance periods (Fig. 5c), except for a decrease in the amygdala that actually stretches into the encoding period. The same observation emerged when distinguishing neuronal subpopulations by their burstiness as characterized by $LvR$ quartiles (Fig. 5d).

We did observe an important modulation of the population burst activity subsequent to the probe presentation ($\sim $0.5 s after; Fig. 5b). The enhanced activity in the entorhinal cortex and amygdala at population level is consistent with the identification of “probe” neurons at single-unit level in Boran et al. (2019); Kamiński et al. (2020). However, this specific neuronal set is not solely responsible for these peaks of activity as they persist, at least partially, even in the absence of probe neurons (Fig. S5). As suggested in the cited works, these activity signals likely indicate a switch between a memory maintenance phase and a retrieval process (Kamiński et al. 2020). This applies especially to the entorhinal cortex. For the amygdala, we observe a minimum in FF that is time-locked to the response time (1 s before) and modulated by higher workloads, which suggests a possible involvement in memory retrieval functions (Fig. 5c and e). The net activity decrease observed in the hippocampal population is however less clear.

#### Final remarks

Summarizing, three main results emerge from the analysis of burstiness, which is a measure of the irregularity of the spike trains, during a WM task in the MTL. First, the promptness of the response, but not the workload, is inversely correlated with burstiness levels (Fig. 4a). The occurrence of these correlations already in the early phases of the task indicates a possible dependence on the global level of attention in the test subjects. Second, probe presentation is characterized by different degrees of burst activity at the population level: strongly enhanced in the entorhinal cortex and amygdala but suppressed in the hippocampus (Fig. 5b). These signals are distributed across the whole population of neurons and may signify a retrieval of information from memory for the amygdala. Third, firing rates associated with non-Poisson (nonrandom) firing, either regular or bursty, can predict better the memory workload than those related to random patterns (Fig. 6). Our results suggest that WM might be maintained through an interplay of heterogeneous spiking dynamics, without clearly favouring either of the proposed models (persistent *vs* transient).

The activities of the regions analyzed here (hippocampus, amygdala, and entorhinal cortex) show distinct features in the spiking patterns and in the association to behavioral and trial data. Overall, they indicate that a broader perspective of the timing and structure of spiking patterns, not constrained to the concept of persistent (enhanced) activity, is needed. In our study, we adopted multiple quantities related to spiking variability, such as $LvR$ (across time), population bursts (across neurons), and Fano factor (across trials), which could be misconceived to be highly correlated when in fact they probe different aspects of the neural code. Indeed, our results suggest that these 3 aforementioned quantities can help in discriminating response times, memory retrieval processes, and workload, depending on the anatomical area. Clearly, the methods utilized here do not exhaust the set of metrics that can offer insights into WM function. For example, classification of spike events or information theory-based metrics can aid our understanding of the simultaneous coding of different signals and how these are shared across neurons (Naud and Sprekeler 2018; Quian Quiroga and Panzeri 2009; Safaai et al. 2018; Williams et al. 2021). In addition, future studies could examine if interactions between different areas also carry information about WM performance (see, e.g. Dimakopoulos et al. (2022)). Also, they should attempt to unveil whether and how the different classes of firing patterns (regular, random, bursty) are generated internally or whether they are induced from a neighboring area (Sreenivasan and D’Esposito 2019). It is likely that heterogeneous references will be more adequate than a simple, standard WM experiment for investigating the discharge patterns. An increased complexity of the tasks, with, e.g. variable length delays, distracting stimuli or different probing schemes (Bausch et al. 2021), but also extended fixation periods could offer more insights into the stability of the spiking sequences.

## Supplementary Material

Supplementary_data_tgac039
