# Supplementary material for: Spiking burstiness and working memory in the human medial temporal lobe
Source: Cereb Cortex Commun. 2022 Oct 19;3(4):tgac039. doi: 10.1093/texcom/tgac039 (PMC9677244; doi:10.1093/texcom/tgac039)
Supplement: Supplementary_data_tgac039 [file supplementary_data_tgac039.pdf]

Spiking burstiness and working memory in the human medial temporal lobe

Supplementary data

Francesco Cocina[1], Andreas Vitalis[1*], and Amedeo Caflisch[1*]

[1] *Biochemistry Department, University of Zurich, Zurich, Switzerland*

[*] Corresponding authors. Email: a.vitalis@bioc.uzh.ch, caflisch@bioc.uzh.ch

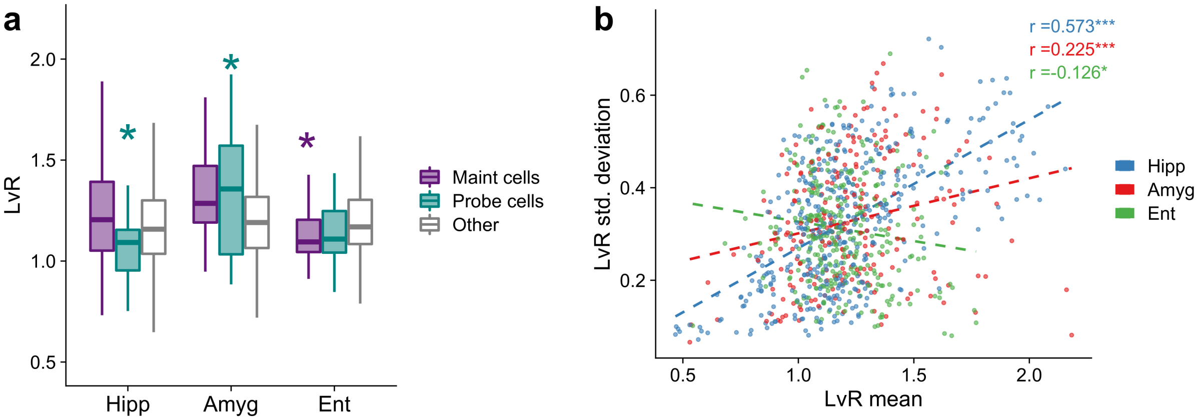

Figure S1: (a) LvR values for maintenance and probe neurons. These were identified following the same procedure as in the original publication. They are characterized by a significant increase of spike counts, relative to the fixation period, in the maintenance and probe periods, respectively. A Wilcoxon rank sum test is performed for both sets of neurons against the remaining units that were not specifically characterized ('Others'). (b) Trial variability of LvR values. Scatter plot of mean and standard deviation computed across trials for each unit. A linear fit is plotted to aid visualization. Pearson correlation values are shown on top with significance levels (Student's t test, $t(416; 231; 304) = (14.2; 3.51; -2.22)$). $*p$ <0.05, ** <0.01, *** <0.001.

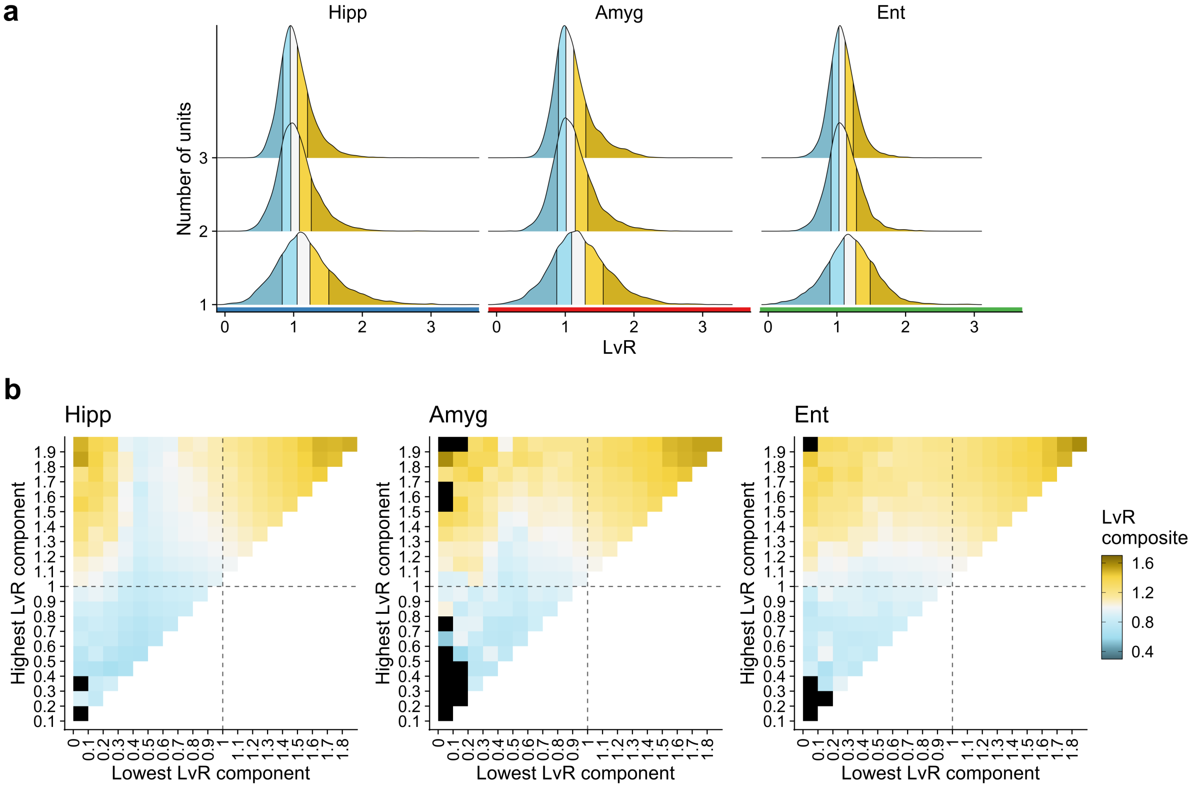

Figure S2: (a) Density distribution of LvR for single units (”1” on y axis) and combinations of these (”2” and ”3”). For the latter, similarly to the results of Fig. 3, we randomly sampled in each session a number of points equal to the number of recorded units. This was done in order to avoid that sessions with the largest number of neurons dominated the overall sample (as the number of combinations increases with the binomial coefficient). Colors indicate the five n-tiles. (b) LvR values resulting from the combination of two spike trains. The x and y axes indicate the values of the constituent single-unit LvR values (threshold at 2); each composite LvR shown is taken as the mean of the values appearing in that particular bin. If the samples are fewer than 5, the average is not computed (black bins). Analogously to the considerations done in panel (a), the averages are performed by weighting such that each session contributes with the effective number of recorded units to the final result rather than with the total number of combinations.

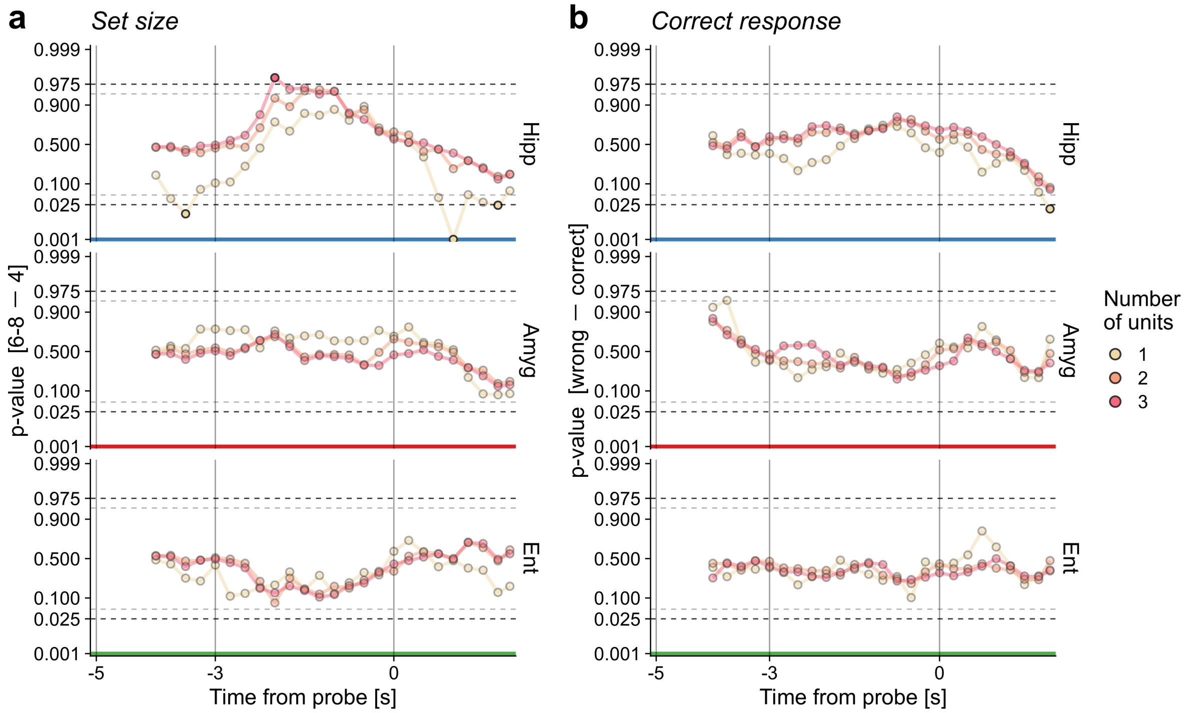

Figure S3: (a) Dependency between set size and burstiness spectrum. To quantify differences between the LvR distributions of set size 6-8 *vs.* 4, we computed a test statistic that measures the signed area between the two cumulative distribution functions, similar to the Wasserstein distance. The calculation follows the same sliding window approach as in Fig. 4. The p values derive from a null distribution generated by shuffling the set size labels but preserving the trial structure; the opacity of symbols denotes significance (dotted lines as two-tailed thresholds corresponding to p value < 0.05). See Fig. S4 for the underlying test statistic values. A probit transformation is applied to the y axis to widen the intervals of significance. (b) Same procedure as in panel (a), but distinguishing correct and wrong responses rather than set sizes.

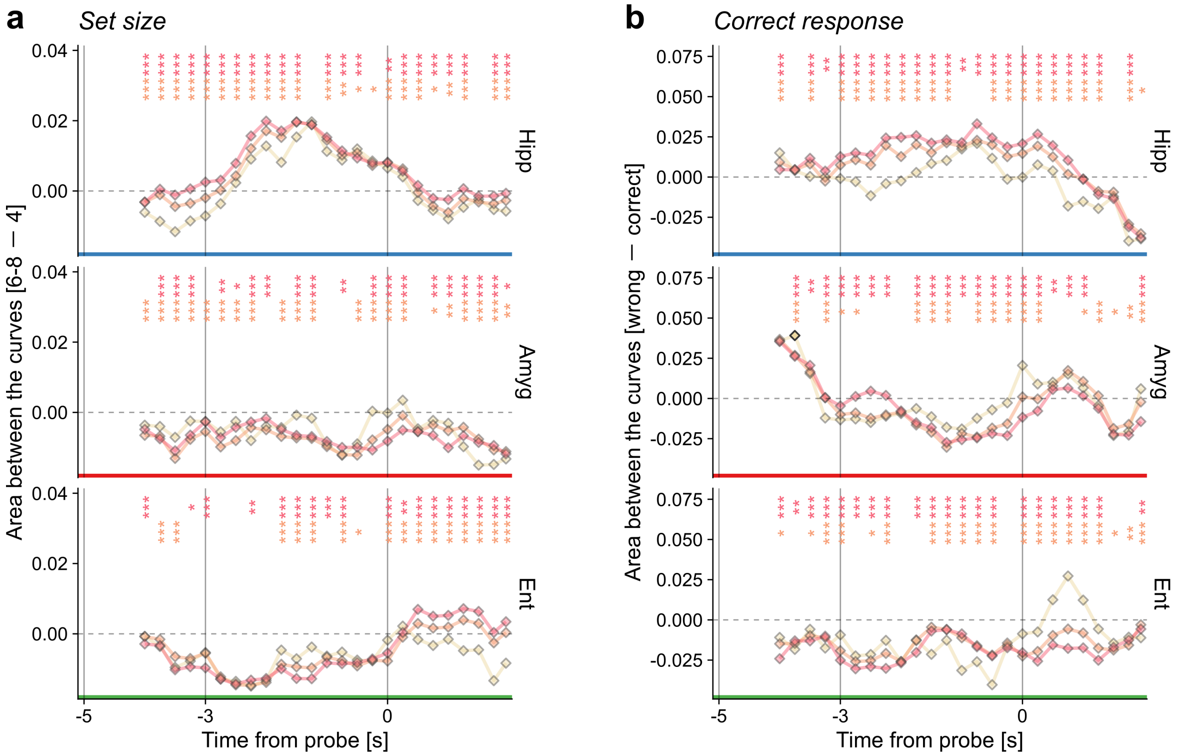

Figure S4: In panels (a) and (b) we show the test statistic values associated with the p values displayed in Figs. S3a-b, respectively. The opacity of the points indicates the significance of single measurements (equivalent to Fig. 4). For each time point, the annotation on top denotes the significance level reached by a t test between the combined measures ("2" or "3", 100 samples each generated by bootstrap, see Sec. 2.3) and the single-unit value, taken as the reference mean of the null hypothesis (*p <0.05, **<0.01, ***<0.001, BH-corrected).

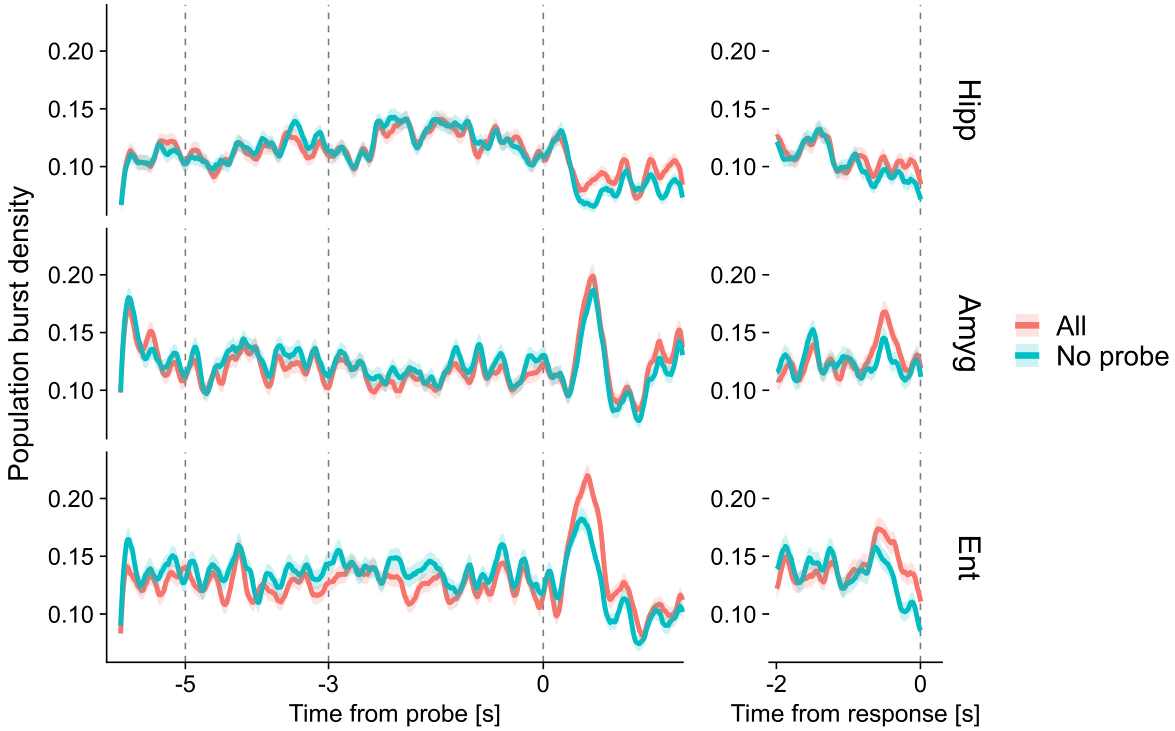

Figure S5: Density of population bursts without probe neurons. Bursts were identified excluding the probe neurons from the neuronal population ('No probe'). The profiles labelled as 'All' are the same as in Fig. 5b

.

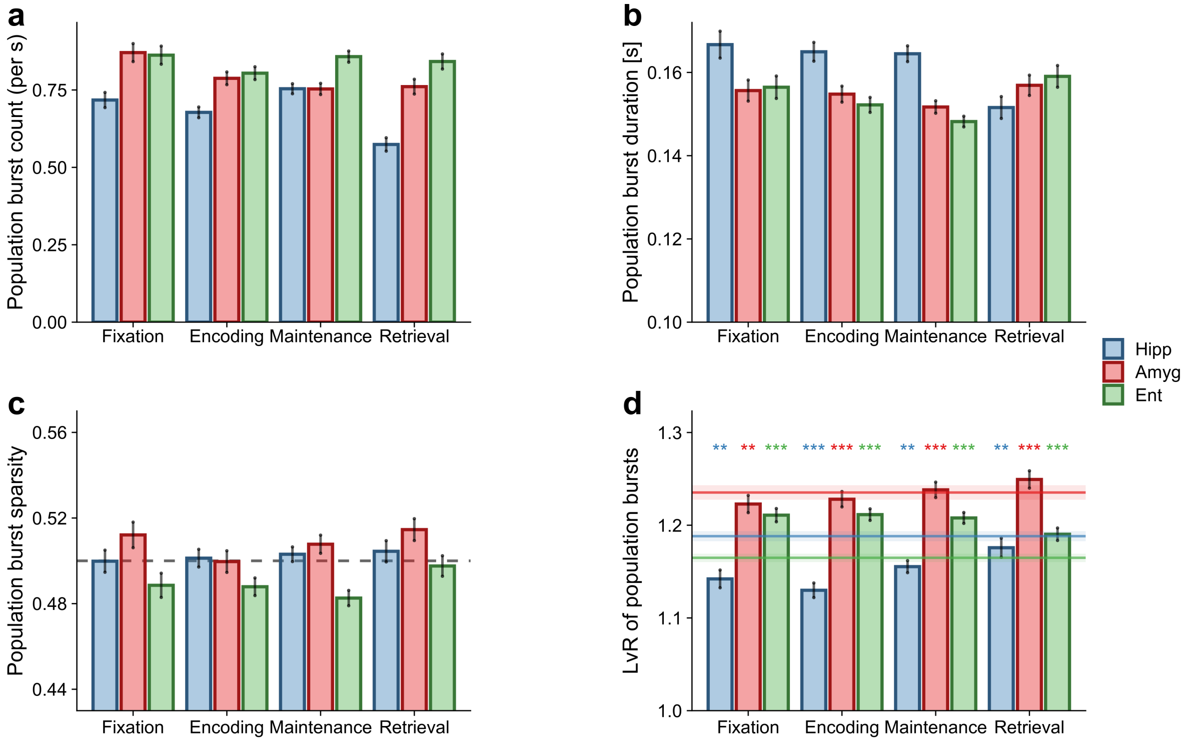

Figure S6: Properties of population bursts. (a) Population burst count. The number of burst events per period was counted within each trial and divided by the respective period lengths. The probe or effective retrieval period is defined only up to the response time. Mean and SEM across all of the pooled trials are shown. Hippocampal measurements tend to be lower everywhere (Kruskal-Wallis test, $p < 0.001$ in all periods; post-hoc pairwise Wilcoxon rank sum tests, $p < 0.001$ everywhere except in maintenance with respect to amygdala). Only hippocampus and amygdala show significant differences across the periods (Kruskal-Wallis test, $p < 0.001$, $= 0.045$, $= 0.27$ for hippocampus, amygdala and entorhinal cortex respectively), most prominently maintenance *vs* retrieval in the hippocampus. (b) Duration of population burst events. Burst events within each trial period were pooled across all of the trials; mean and SEM of the burst lengths are shown. In (a) and (b), trials with response times higher than 2 seconds were discarded. Hippocampal bursts are significantly longer than those of amygdala and entorhinal cortex during the maintenance period (Kruskal-Wallis $p < 0.001$, post-hoc Wilcoxon tests all $p < 0.01$) and become shorter in the retrieval epoch when compared to the other periods (Kruskal-Wallis $p < 0.001$, post-hoc Wilcoxon tests all $p < 0.01$). (c) Sparsity of the burst unit components. Sparsity values quantify the degree of contribution of unit activities to the overall firing rate during bursts. Low values indicate that only few units are active during the burst whereas higher values point to a balanced contribution of a large fraction of the units (see details in Sec. 2.4). We computed averages within each trial and report here the mean and SEM across the whole pool of trials. (d) Burstiness of the contributing units. We computed for each burst a LvR value by taking the weighted mean of single-unit LvR values. The weights were proportional to the firing rates during the burst event window (unit contributions, see details in Sec. 2.4). Horizontal lines indicate the average LvR values of single units. The plotted values are the mean and SEM of trial-averaged LvR values of bursts computed. Wilcoxon signed rank tests were performed between burst and single-unit results for each combination of anatomical area and trial period (n=680, 607, 632 average number of trials for hippocampus, amygdala, and entorhinal cortex, respectively; *p <0.05, **<0.01, ***<0.001, BH-corrected). Hippocampal bursts tend to be composed of more regular units: weighted values are lower (Wilcoxon signed rank test, p values annotated on top); amygdalar are consistent in burstiness with average single-unit behavior; finally, the entorhinal cortex exhibits a preference for burstier neurons (weighted values are higher).

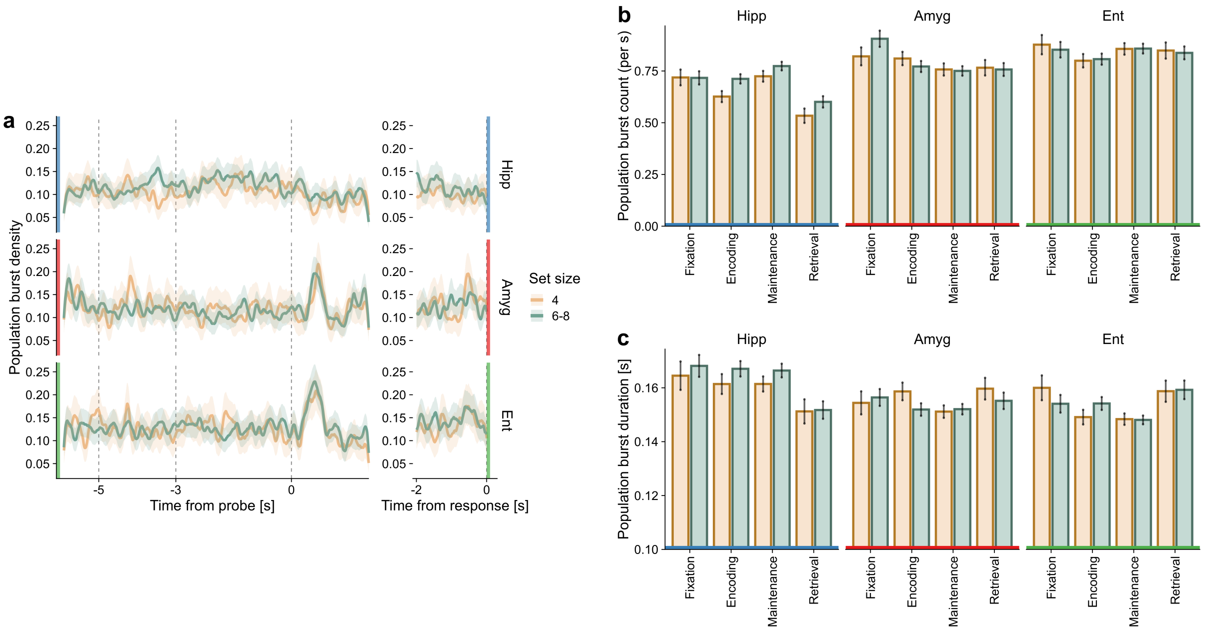

Figure S7: Population burst results for different set sizes. The analyses shown in panels (a)-(c) correspond to those presented in Figs. 5b and S6a-b, respectively.

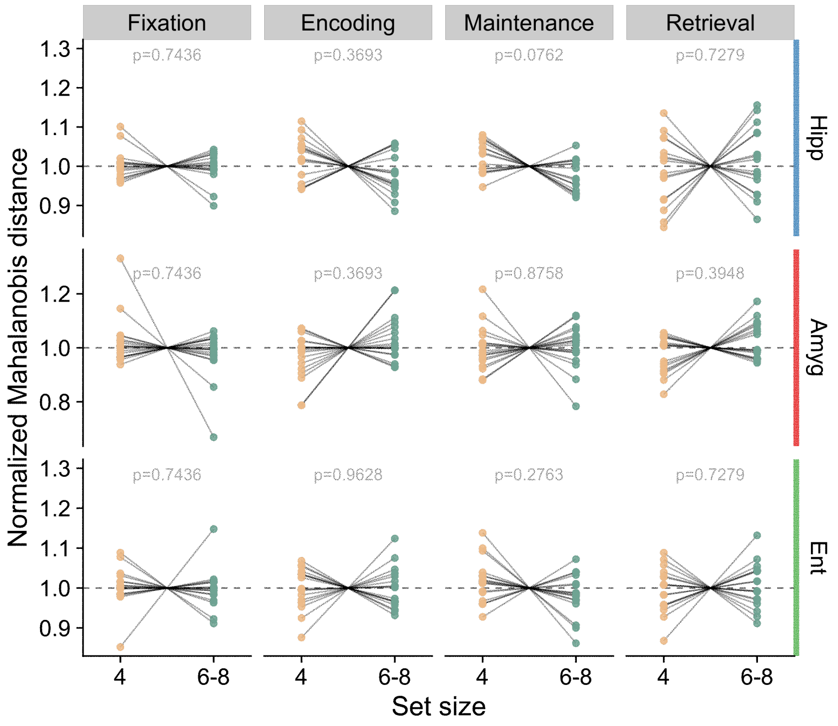

Figure S8: Distance between burst compositions of different set sizes. Within each session, the Mahalanobis distance was calculated between single bursts and those of a reference group - in this case those in the fixation period - by using the unit composition of the bursts as the metric space (see Sec. 2.4). In order to account for the intrinsic (*i.e.,* workload-blind and trial-wise) variability of the burst composition, we properly rescaled each unit dimension such that high firing rates, with higher deviations, do not dominate the calculation. For each anatomical region we selected those sessions with at least five recorded units and ten bursts overall during the fixation periods. Eventually, we averaged across the single $M(\boldsymbol{w}_i)$ within each combination of trial period and set size. Here, we display the average values of Mahalanobis distances that were obtained for both of the set size classes and, afterwards, normalized by the sum of the two. p values are shown on top of each sub-panel (Wilcoxon signed rank or t tests, $n = 16$, 18 and 17 for hippocampus, amygdala, and entorhinal cortex, respectively; BH correction across anatomical areas).

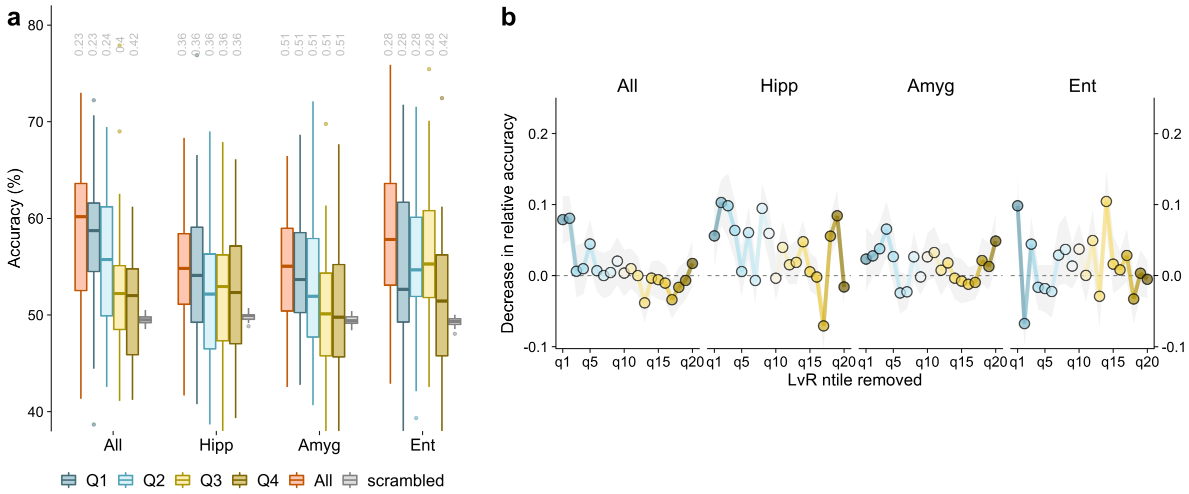

Figure S9: Decoding of correct response from population activity during maintenance. The analyses shown in panels (a)-(b) correspond to those presented in Figs. 6a-b, respectively.
